# Survival, toxicity and length of stay after haploidentical or cord blood transplantation in a Latin American center: a cross-sectional, comparative study

**DOI:** 10.1038/bcj.2017.44

**Published:** 2017-05-26

**Authors:** I Esteves, F P S Santos, A A F Ribeiro, A T Kondo, J F Fernandes, F R Kerbauy, L Kerbauy, N Hamerschlak

**Affiliations:** 1 Oncology and Hematology Center, Hospital Israelita Albert Einstein, São Paulo, Brazil; 2 Department of Bone Marrow Transplantation, Hospital Israelita Albert Einstein, São Paulo, Brazil; 3 Oncology and Hematology Center, Hospital Israelita Albert Einstein, São Paulo, Brazil; 4 Blood Bank, Hospital Israelita Albert Einstein, São Paulo, Brazil; 5 Department of Bone Marrow Transplantation, Hospital Israelita Albert Einstein, São Paulo, Brazil; 6 Hematology and Bone Marrow Transplantation Program, Hospital Israelita Albert Einstein, São Paulo, Brazil; 7 Department of Marrow Transplantation, Hospital Israelita Albert Einstein, São Paulo, Brazil; 8 Hematology and Bone Marrow Transplantation, Albert Einstein Hospital, São Paulo, Brazil

Only a minority of patients find a fully compatible donor for hematopoietic stem cell transplantation (HSCT). In the absence of human leukocyte antigen-matched related or unrelated donors, haploidentical donors (HAPLO) and umbilical cord blood and placental (UCB) cells are alternatives for patients with malignant and non-malignant hematological diseases. However, the risk of graft-versus-host disease (GvHD) is high for both options, requiring more intensive immunosuppression to overcome a higher degree of human leukocyte antigen mismatch.^1, 2^

We compared clinical outcomes, length of hospital stay (LOS) and survival of patients receiving HSCT from HAPLO with those receiving it from UCB cells over a period of 5 years in a major private tertiary hospital in Brazil—something that few studies did.^3, 4^ In a retrospective, comparative study, we included all consecutive patients undergoing HSCT without an human leukocyte antigen-matched donor during a period of 5 years. We reviewed all electronic medical records of transplanted from October 2007 to October 2012, and divided cases according to the source of cells: UCB or HAPLO. All patients signed informed consent forms for treatment and permission was obtained from the Institutional Review Board to access the charts and analyze the data.

We evaluated, as a primary end point, the overall and progression-free survival from the day of hematopoietic stem cells infusion to the last day of follow-up or death, and as secondary end points, LOS, treatment-related mortality, relapse rate and the development of other transplant-related toxicities (oral mucositis, sinusoidal obstructive syndrome (SOS), GvHD (grade), cytomegalovirus (CMV) reactivation, graft failure). Any other hospital admission during the first year post-transplantation was also evaluated, as were baseline general demographic and clinical variables. After transplantation, cases were evaluated according to engraftment, oral mucositis grade, SOS, acute or chronic GvHD, CMV reactivation, death and cause of death.

Neutrophil engraftment was defined when neutrophil serum levels were higher than 0.5 × 109/l for three consecutive days. Graft failure was considered when the patients did not achieve this level by day +45 post transplant. Chronic and acute GvHD were defined according to already established criteria.^5^ Classification of conditioning regimens as myeloablative, non-myeloablative and reduced intensity regimens was based on the literature^6, 7^ Hepatic SOS was defined according to the Seattle and Baltimore modified criteria.^8, 9^ CMV reactivation was defined with serum PCR tests showing elevated copy numbers (above 214 copies). Underlying disease recurrence was considered for patients with malignant disorders and diagnostic criteria for this were computed tomography and/or positron emission tomography-positive for lymphoproliferative diseases and histology confirmation. For other hematological malignancies, such as acute leukemias, bone marrow evaluation and immunophenotyping confirming relapse were decisive.

For the statistical analysis, we used descriptive measures, Kaplan–Meier tests log rank test and univariate Cox models to verify the odds ratios. The probability of clinical toxicities (such as engraftment failure, SOS, acute or chronic GvHD and relapse) was analyzed as cumulative incidence over time. Groups were compared using log-rank test. All variables significant at *P*<0.10 in the univariate analysis were included in the stepwise multivariate analysis model. LOS was assessed by the Mann–Whitney *U-*test using medians.

In the study period, 92 patients were admitted for HSCT. Out of the 92 patients, 50 received cells from UCB and 42 from HAPLO. The groups were homogeneous regarding baseline demographic and clinical variables (Table 1). A previous allogeneic transplant was significantly more frequent in the HAPLO group (*P*=0.039) and the myeloablative regimen was the most frequent in the UCB group (48%), consisting of busulfan/fludarabin/thiotepa, with reduced toxicity regimens based on busulfan/fludarabine. GvHD prophylaxis was also different between groups (Table 1). The source of hematopoietic stem cells for the HAPLO group was the bone marrow for 92% of the patients, and 8% received peripheral blood cells.

Follow-up time for all patients was 179 days (median) varying from 12 to 2333 days (interquartile range: 74–517); with 160 days for the UCB group (range: 12–2183 days; interquartile range: 67–796 days) and 196 days for the HAPLO group (range: 28–2333 days; interquartile range of 104–381 days). It was not possible to calculate median survival time for the survivors only, because there were <50% of deaths in all groups until the end of study.

Oral mucositis prevalence and grade were similar between groups (Table 1), as were graft failure and CMV reactivation up to D+100, SOS and the prevalence of chronic and acute GvHD and the number of deaths were similar too. Infection was the main cause of death. SOS grade was significantly associated with mortality (*P*=0.004; log-rank test) in the UCB group. Acute GvHD was associated with risk of death (*P*=0.009; log-rank test), with significant differences between grades (*P*<0.001; log-rank test). The prevalence of acute GvHD in 100 days was 44.9% in the UCB group (confidence interval (CI) 30.5–58.3%), and in 1 year, 46.9% (CI 32.3–60.3%). Recurrence of malignant diseases up to day+100 occurred in 4% (CI 0.72–12.3%), and in 1 year, 4% (CI 0.72–12.2%).

The male sex (*P*=0.036) and the myeloablative regimen (*P*=0.033) were significantly associated with mortality in the UCB group and the multivariate analysis confirmed the correlation of gender and death (log rank; *P*=0.044; Table 2).

Mortality was higher in patients 19–59 years old receiving HAPLO (Table 2). There was no difference between groups regarding conditioning regimen, GvHD prophylaxis, SOS, GvHD, graft failure, ABO incompatibility, CMV reactivations, causes of death or relapse rate. In the multivariate analysis, age above 60 years significantly affected the survival of patients undergoing HSCT from haploidentical donor (log-rank=0.024).

Considering death as a competitive event, the cumulative incidence of acute GvHD in the HAPLO group up to D+100 was HR 21.4% (CI 10.4–34.9%) and the incidence of chronic GvHD in 1 year after HSCT was 7.1% (CI 1.8–17.7%). The incidence of malignant disease recurrence up to D+100 was 9.5% (CI 2.98–20.7%), and in 1 year, 19% (CI 8.7–32%). The higher the number of hospital admissions in the first year after HSCT, the higher the survival (*P*=0.012; Table 2). Patients over 60 years old had a higher risk of death and this was confirmed by the Kaplan–Meier (log rank; *P*=0.036) curve.

In the UCB group, LOS during HSCT was 67 days (40.25–77.75), and the median number of days in hospital during the first year after HSCT (that is, in additional admissions) was 9.50 (0.00–23.75). The number of hospital admissions in the first year was 1.50 (0.00–3.00). The HAPLO group had a median LOS in the first year after HSCT of 45.00 days (18.00–83.00). LOS during HSCT was 51 days (36.75–78.00) and the number of hospital admissions in the first year after HSCT was 2.00 (1.00–3.00). In this study, the higher the number of hospital admissions in the first year after HSCT, the higher was the survival for both groups (Table 2). This finding is probably related to the faster management of HSCT complications in the hospital, a proper care that is naturally reflected in the survival rates. As a limitation of any retrospective study based on medical charts review, it was not possible to identify what characteristics, in addition to the conditioning regimen, would directly influence survival rates.

Previous studies have indicated that conditioning regimen, degree of human leukocyte antigen mismatch, incidence of acute and chronic GvHD, graft failure and infection can impact on mortality.^10^ In fact, our results have also shown that with severe degrees of SOS and acute GvHD impair survival, but this happened with HSCT from UCB only.

Disease relapse has been pointed out as a cause of death in patients receiving HSCT from UCB with myeloablative regimen,^2^ comprising 13 to 41% of cases of malignant diseases.^11, 12^ In this survey, it was 19% in patients receiving HSCT from UCB. Adequate selection of patients is important to reduce these rates. Myeloablative regimens are risk factors for SOS,^13^ and the patients in our study who died from SOS had indeed received myeloablative regimens.

Haploidentical HSCT has become possible through the improvements of the techniques of T-cell depletion, immunosuppression after HSCT and reduced intensity of the conditioning regimen.^14^ However, they are generally high-cost procedures due to clinical complications and prolonged hospital stay.^15^ HSCT with alternative sources has a high cost,^3^ and the UCB source can be even more expensive than the haploidentical stem cell source.^16^ This study has not evaluated direct or indirect costs, but LOS, a cost component, was assessed in the medical records, showing no significant differences between groups.

We confirmed our hypothesis that UCB, as much as the HAPLO source, can be viable and safe sources of hematopoietic stem cells, although toxicity events in UCB might have had a higher impact on survival than in HAPLO. Both sources can be considered options *in situations* where the patient is in imminent danger of death by the absence of fully compatible donors.

## Figures and Tables

**Table 1:** Demographic and clinical characteristics of patients treated with HSCT from HAPLO or UCB in Brazil, 2007–2012; values expressed as medians (s.d.)

*Variables*	*HAPLO* n=*42*	*UCB* n=*50*	*Total*	P*-value*
	n *(%)*	n *(%)*	n *(%)*	
*Baseline data*				
*Age (years)*				
0–18	19 (45.2)	34 (68)	53 (57.6)	0.055
19–59	20 (47.6)	12 (24)	32 (34.7)	
>60	3 (7.1)	4 (8)	7(7.6)	
*Sex*				
Female	15 (35.7)	17 (34)	32 (31.6)	0.999
Male	27 (64.3)	33 (66)	60 (68.4)	
ABO-incompatible	22 (52.4)	32 (63.3)	54 (58.7)	0.291
ABO-compatible	20 (47.6)	18 (36.7)	38 (41.3)	
* Diagnosis and disease status*				
Non-malignant diseases	23 (54.8)	19 (38)	42 (54.3)	0.142
Malignant diseases	19 (45.2)	31 (62)	50 (45.6)	
Previous HSCT	8 (19)	2 (4)	18 (24)	**0.039**
First complete remission	25 (59.5)	25 (51)	50 (54.3)	
Myeloablative regimen	13 (31)	24 (48)	37 (40.2)	**<0.001**
Non-myeloablative regimen	24 (57.1)	6 (12)	30 (32.6)	
Reduced toxicity regimen	5 (11.9)	20 (40)	25 (17.4)	
Graft failure	6 (14.3)	10 (20)	16 (17.4)	0.585
Engraftment	36 (85.7)	40 (80)	76 (82.6)	
MMF+calcineurin inhibitor	0 (0.0)	34 (68.0)	34 (36.9)	**<0.001**
MTX+calcineurin inhibitor	0 (0.0)	3 (6.0)	3 (3.6)	
Steroids+calcineurin inhibitor	3 (7.1)	13 (26.0)	16 (17.4)	
Cyclophosphamide	36 (85.7)	0 (0.0)	36 (39.1)	
T-cell depletion protocol	3 (7.1)	0 (0.0)	3 (17.4)	
				
*Toxicity*				
No oral mucositis	22 (52.4)	22 (44)	44 (47.8)	0.530
Oral mucositis	20 (47.6)	28 (56)	48 (52.1)	
Mucositis grades I and II	14 (68.7)	23 (83.3)	37 (40.2)	0.051
Mucositis grades III and IV	6 (31.3)	5 (16.6)	11 (12)	
No SOS	40 (95.2)	42 (84)	82 (88.2)	0.103
SOS	2 (4.8)	8 (16)	10 (11.8)	
Mild SOS	0 (0)	1 (12.5)	1 (1.08)	0.533
Moderate SOS	1 (50)	6 (75)	7 (7.6)	
Severe SOS	1 (50)	1 (12.5)	2 (2.17)	
Acute GvHD	12 (28.6)	23 (46)	35 (38)	0.131^a^
Acute GvHD grade 1	0 (0)	3 (13)	3 (3.2)	0.509
Acute GvHD grade 2	5 (42.9)	11 (47.8)	16 (17.4)	
Acute GvHD grade 3	3 (28.6)	6 (26.1)	9 (9.8)	
Acute GvHD grade 4	3 (28.6)	3 (13)	6 (6.5)	
Chronic GvHD	4 (9.5)	4 (8)	8 (8.8)	0.999^b^
CMV reactivation	30 (71.4)	35 (70)	65 (68.4)	0.818^c^
Relapse	8 (19)	2 (4)	10 (11)	**0.039**^d^
Death	11 (26.2)	23 (46)	34 (36.9)	0.055
Cause of death: infection	7 (63.6)	17 (73.9)	24 (26)	0.675
Cause of death: relapse	3 (27.3)	2 (8.7)	5 (5.4)	
Cause of death: GvHD	0 (0)	2 (8.7)	2 (2.2)	
Other causes of death	1 (9.1)	2 (8.7)	3 (3.2)	

Abbreviations: CMV reactivation, cytomegalovirus infection reactivation up to the Day+100 after HSCT; GvHD, graft-versus host disease; HAPLO, haploidentical donors; HSCT, hematopoietic stem cell transplantation; MMF, mycophenolate mofetil; MTX, metothrexate; SOS, sinusoidal obstructive syndrome; UCB, umbilical cord/placental blood.

a Versus no acute GvHD.

b Versus no chronic GvHD.

c Versus no CMV activation.

d Versus no relapse. Bold values are significant associations (*P*<0.05).

**Table 2:** Correlation (univariate Cox analysis) between demographic and clinical variables and survival in patients undergoing HSCT with cells from UCB and from HAPLO

	*Coefficient*	*HR*	*CI*		P*-value*
			*Inferior*	*Superior*	
Cellularity (TNC) in UCB	0.00	1.00	0.14	7.10	0.629
Cellularity (TNC) in HAPLO	0.00	1.00	2.72	2.72	0.961
Hospital admissions in the first year in UCB	−0.94	0.39	0.18	0.84	**<0.001**
Hospital admissions in the first year in HAPLO	−0.69	0.50	0.97	2.83	**0.012**
Male gender in UCB	1.11	3.04	0.01	1188.58	**0.044**
Male gender in HAPLO	0.06	1.06	0.85	9.93	0.921
					
*Age in UCB*					
19–59	0.05	1.05	0.13	8.16	0.924
> 60	0.19	1.21	0.11	12.86	0.803
					
*Age in HAPLO*					
19–59	0.56	1.76	1.38	24.32	0.441
> 60	1.87	6.48	129.03	3297.48	**0.024**
Diagnosis in UCB: malignant disorder	0.44	1.55	0.07	31.99	0.338
Diagnosis in HAPLO: malignant disorder	1.23	3.41	8.04	114.50	0.070
					
*ABO incompatibility in UCB*					
No	−0.37	0.69	0.18	2.68	0.440
					
*ABO incompatibility in HAPLO*					
No	−0.66	0.52	0.49	5.75	0.293
					
*Prophylaxis* *GvHD* *in UCB*					
MTX+calcineurin inhibitor	−18.31	0.00	0.00	0.00	0.998
Steroids+calcineurin inhibitor	−0.50	0.61	0.18	1.99	0.323
					
*Conditioning regimen in HAPLO*					
Reduced intensive	−0.46	0.63	0.23	15.27	0.665
Myeloablative	−0.21	0.81	0.58	8.72	0.762
Chronic GvHD in UCB (yes)	−18.22	0.00	0.00	0.00	0.997
CMV in UCB (yes)	−0.58	0.56	0.19	1.67	0.186
CMV in HAPLO (yes)	1.15	3.15	2.97	181.90	0.275
LOS during HSCT in UCB	0.00	1.00	0.14	7.10	0.989
LOS during HSCT in HAPLO	0.00	1.00	2.69	2.76	0.566
LOS in the first year after HSCT in UCB	−0.01	0.99	0.14	6.88	0.165
LOS in the first year after HSCT in HAPLO	0.01	1.01	2.72	2.76	0.080
Relapse in UCB (yes)	0.92	2.52	0.02	348.06	0.219
Relapse in HAPLO (yes)	0.21	1.24	0.74	16.06	0.787
Cellularity (CD34+) in UCB	0.00	1.00	0.14	7.10	0.660
Cellularity (CD34+) in HAPLO	0.00	1.00	2.72	2.72	0.590
Mucositis in UCB (yes)	0.42	1.52	0.08	29.86	0.340
Mucositis in HAPLO (yes)	0.17	1.19	1.00	10.75	0.779
Acute GvHD in UCB (yes)	−1.12	0.32	0.17	0.61	**0.014**
Acute GvHD in HAPLO (yes)	−0.23	0.79	0.58	8.34	0.730

Abbreviations: CI, confidence interval; CMV reactivation, cytomegalovirus infection; GvHD, graft-versus host disease; HAPLO, haploidentical donors; HR, hazards ratio; HSCT, hematopoietic stem cell transplantation; LOS, length of hospital stay; MMF, mycophenolate mofetil; MTX, metothrexate; SOS, sinusoidal obstructive syndrome; TNC, total nucleated cell count; UCB, umbilical cord/placental blood. Bold values are significant associations (*P*<0.05).
